# Non-destructive mapping of grain orientations in 3D by laboratory X-ray microscopy

**DOI:** 10.1038/srep14665

**Published:** 2015-10-23

**Authors:** S. A. McDonald, P. Reischig, C. Holzner, E. M. Lauridsen, P. J. Withers, A. P. Merkle, M. Feser

**Affiliations:** 1 Manchester X-ray Imaging Facility, School of Materials, University of Manchester, Manchester, M13 9PL, UK; 2 Xnovo Technology ApS, Galoche Alle 15, 4600 Køge, Denmark; 3 Carl Zeiss X-ray Microscopy, Inc., 4385 Hopyard Road, Suite 100, Pleasanton, CA 94588, USA

## Abstract

The ability to characterise crystallographic microstructure, non-destructively and in three-dimensions, is a powerful tool for understanding many aspects related to damage and deformation mechanisms in polycrystalline materials. To this end, the technique of X-ray diffraction contrast tomography (DCT) using monochromatic synchrotron and polychromatic laboratory X-ray sources has been shown to be capable of mapping crystal grains and their orientations non-destructively in 3D. Here we describe a novel laboratory-based X-ray DCT modality (LabDCT), enabling the wider accessibility of the DCT technique for routine use and in-depth studies of, for example, temporal changes in crystallographic grain structure non-destructively over time through ‘4D’ *in situ* time-lapse studies. The capability of the technique is demonstrated by studying a titanium alloy (Ti-β21S) sample. In the current implementation the smallest grains that can be reliably detected are around 40 μm. The individual grain locations and orientations are reconstructed using the LabDCT method and the results are validated against independent measurements from phase contrast tomography and electron backscatter diffraction respectively. Application of the technique promises to provide important insights related to the roles of recrystallization and grain growth on materials properties as well as supporting 3D polycrystalline modelling of materials performance.

The majority of metallic and ceramic engineering materials of interest are polycrystalline. Their properties are strongly influenced by grain-scale effects relating to the orientation, size and shape of individual grains or clusters of grains. The electron backscatter diffraction (EBSD) technique^1^,^2^ has, since its introduction in the 1980 s, provided detailed grain orientation maps in 2D at the surface. However, EBSD can only be extended to the third dimension by destructive serial sectioning^3^,^4^ and is only suited for relatively small volumes. Studying temporal changes within the bulk of one and the same sample, such as the dynamics/kinetics of the grain growth process for example, is thus precluded. Only measurements of separate samples representing different evolution steps are possible with 3D-EBSD. This makes direct interpretation very difficult since the evolution of the grain structure can’t be followed directly. Several techniques to allow mapping of crystal orientation in 3D have been developed in recent years, for the most part using monochromatic X-ray beams of very high flux found at 3^rd^ generation synchrotron sources. These include X-ray diffraction contrast tomography (DCT)^5^,^6^,^7^,^8^,^9^ and 3D X-ray diffraction microscopy (3DXRD)^10^,^11^. Techniques using a point focused, polychromatic X-ray beam include differential aperture X-ray microscopy (DAXM)^12^,^13^. The DCT technique, distinguished from each of these other methods, has the advantage that access to both the three-dimensional crystallographic information *and* the sample’s microstructure is provided. It has enabled the study of the relationship between crystallographic microstructure and material behaviour during initiation of damage, such as the interaction between intergranular stress corrosion cracking and microstructure^14^, and the shape and orientation of grains around short fatigue cracks^15^,^16^ as well as temporal information about grain growth and reorientation taking place during sintering^17^.

Clearly, given the much wider availability and accessibility of laboratory X-ray microtomography systems, the development of a laboratory-based DCT technique is an attractive prospect. The first results of three-dimensional grain mapping in the laboratory has been presented in^18^ for a sample with relatively large (170 μm) and few grains, utilizing an intricate indexing approach based on identifying paths of diffraction spots as they precess across the detector as the sample rotates. In this paper, we present first application results of a novel laboratory-based X-ray DCT modality using a Laue focusing approach, enabling mapping of grains and their crystallographic orientation in 3D within the bulk of polycrystalline materials^19^. The LabDCT technique is implemented as an optional imaging module on a commercially available X-ray microscope (ZEISS Xradia 520 Versa with DCT reconstruction software Xnovo GrainMapper3D). It allows using the same laboratory instrument for conventional absorption- and propagation phase contrast-based X-ray tomography measurements and DCT measurements. The X-ray source produces a divergent polychromatic X-ray beam, as opposed to the parallel beam typically used in the synchrotron X-ray DCT technique. The method is exemplified in this paper through a study of a beta titanium alloy (Tiβ21S) in which an alpha phase is precipitated on the grain boundaries^20^, thus revealing the 3D grain shapes through different material density. Validation of the spatial location of grains measured by LabDCT is performed, while grain orientations are compared to EBSD analysis of a cross-section of grains in the sample. Finally, the wider applications of the LabDCT technique are considered.

## Results

### Setup and implementation

**Figure 1:**
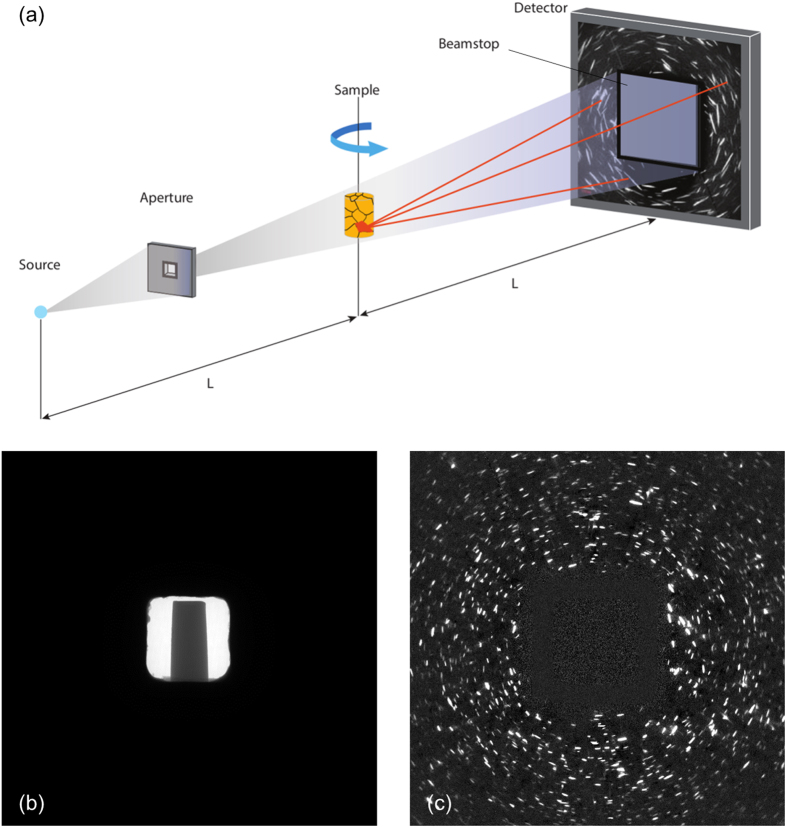
(**a**) Schematic showing the experimental setup of LabDCT in the laboratory x-ray microscope. (**b**) Example absorption contrast projection with the direct beam illuminating the sample in the central region of the detector. (**c**) Image showing diffraction spots from grains within the sample at a single rotational position. Note that the direct beam is blocked by a beam stop.

The instrument uses a micrometer spot size laboratory transmission-type X-ray source with cone beam geometry and a high-resolution optically-coupled detector able to detect the X-rays transmitted through and diffracted by the sample; see Fig. 1(a) for an illustration of the experimental setup. The X-ray source produces a diverging ‘quasi-white’ or broadband beam of X-rays, i.e. a beam with a wide wavelength spectrum (continuum Bremstrahlung from a Tungsten target). While characteristic emission lines of W are present in the spectrum, they don’t contribute strongly to the signal and can be neglected. When a polycrystalline material (in our case a 0.3 mm diameter cylinder of Ti-β21S) is illuminated by the X-ray beam each of the crystal grains within the sample diffracts the beam such that a diffraction pattern is formed on the detector. Diffraction reflections manifest themselves at specific scattering angles (2*θ*) corresponding to the lattice spacing (*d*) and orientation of the crystal planes and the specific X-ray energy (or narrow range; with wavelength *λ*) that is selected from the incoming X-ray spectrum to fulfill the Bragg condition for reflection (*λ *= 2*d*⋅sin*θ*). With a polychromatic X-ray spectrum diffraction events are not observed at discrete angles; instead different energies are diffracted at varying diffraction angles and each grain may contribute multiple reflections to a single diffraction pattern relaxing the requirement on the number of projections needed for the grain reconstruction, therefore constraining the overall acquisition times needed for the LabDCT method, which are comparable to typical absorption tomography acquisition periods.

The current approach takes advantage of the fact that for a point X-ray source with a divergent beam a crystal grain diffracts X-rays such that they are focused in the plane of diffraction at a distance equal to the source-sample distance^21^. The detector is placed at this distance (in the Laue focal plane), which results in a geometric magnification of zero in the plane of diffraction. The pattern of the diffracted beam generally forms a narrow line in the Laue focal plane since the focusing only occurs in the diffraction plane and the beam divergence remains unchanged in the perpendicular plane. Thus, the diffracted signals appear as line-shaped spots (see Fig. 1(c)). Each line-shaped spot in the diffraction pattern originates from the diffraction and focusing of X-rays from one crystallographic lattice plane within one crystal grain of the sample. The focusing effect is a result of symmetry and enabled by the fact that different energies or wavelengths within the X-ray beam meet the Bragg condition at different positions over the extent of the crystal grain. The length of a line-shaped spot is a projected representation of the diffracting grain’s physical size, in the plane perpendicular to the diffraction, magnified by a factor equal to the geometric magnification of the setup. For equal source to sample and sample to detector distance L, see Fig. 1(a), the geometric magnification is equal to two. A high-resolution detector is therefore required to be able to resolve grain dimensions in the Laue focal plane at the micro-metre scale.

In order to collect the required diffraction information, two additional elements are introduced to the setup of the standard X-ray microscope. Firstly, an aperture is placed between the source and sample, which restricts the size of the direct X-ray beam, illuminating the sample only in the central region of the detector and leaving the outer part dark as shown in Fig. 1(b). The ability to use different size apertures allows the number of grains illuminated by the X-ray beam to be adjusted. This is advantageous in managing the number of diffraction spots in the images and to control their degree of overlap. Secondly, a beam stop is used to attenuate the direct X-ray beam transmitted through the sample in order to be able to collect high fidelity diffraction signals.

For LabDCT measurements two consecutive scans are performed on a ZEISS Xradia 520 Versa microscope equipped with a GrainMapper3D™ analysis package developed by Xnovo Technology. The first scan collects the absorption contrast projections in the direct beam. These images are used to produce an absorption contrast reconstruction of the sample. The second scan collects the diffraction patterns using a diffraction optimized detector with beam stop, an example image of which is shown in Fig. 1(c). Grains diffract the beam on to the outer part of the detector. These images are used to calculate the orientations of grains and their positions in relation to the sample reconstruction obtained from the first scan.

### Data processing

A grain or crystal lattice orientation is described as a rotation relative to a reference crystal orientation in the sample, and is quantified by three independent parameters, typically three Euler angles or a Rodrigues vector. The possible range of these parameters is referred to as the orientation space and is restricted by the crystal symmetry. The higher the crystal symmetry, the smaller the orientation space, and it is smallest for cubic crystals.

Grain maps of polycrystals with one or more known phases are represented in two common forms:

- a) A distribution of the local orientation over a three-dimensional grid (voxellated volume), showing grain shapes and positions. The task is to find the local orientation for each grid point, that is 3 parameters.
- b) A list of individual grains, their centroid position (3 parameters), mean orientation (3 parameters) and volume.

Using a polychromatic, conical X-ray beam and irradiating a relatively large 3D sample gauge volume enables an efficient use of the photons generated in a laboratory-based X-ray source, however, it poses the following challenges for the data processing:

- 1) Large 3D sample gauge volume: The solution space is also large, as the diffraction signal may originate from anywhere within this region. Many grains may diffract simultaneously which increases the possibility of diffraction spot overlap.
- 2) Divergent conical beam: The local incident beam direction varies across the irradiated sample volume, and the flux density of the beam drops rapidly at larger source-sample distances.
- 3) Polychromatic beam: Higher number of {*hkl*} lattice plane families diffract simultaneously, increasing the possibility of diffraction spot overlap. The photon energy, Bragg angle or {*hkl*} family of the measured diffraction spot is not known.

The computational challenge comes from the fact that the solution space is large both in real space and in orientation space. Conventional tomographic reconstruction algorithms alone cannot be applied because the projection (diffraction) geometry is not known initially. Indexing and shape reconstruction algorithms that associate the observed diffraction spots with their grains of origin applying spatial and crystallographic criteria have been developed for synchrotron-based 3DXRD^10^,^22^,^23^ and DCT^5^,^6^. More recently, successful indexing and shape reconstruction approaches were demonstrated for a laboratory-based DCT method for samples containing a limited number of grains^18^.

The developed GrainMapper3D™ analysis package uses a proprietary data processing algorithm that allows for obtaining grain centroids of several hundreds of grains from a single scan with a small number of projections, over a range of different grain sizes. The current version of the GrainMapper3D™ software provides the centroid positions, volumes and crystallographic orientations of grains with a known crystallographic phase. Statistical measures are given about the quality and confidence level of the grains and the reconstruction. The resulting grain map is aligned with the absorption reconstruction of the sample.

**Figure 2:**
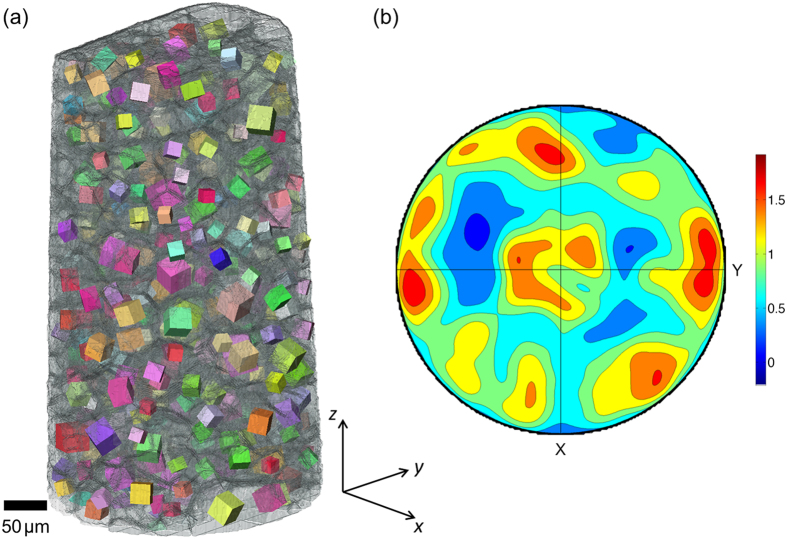
(**a**) Phase contrast reconstruction of the Ti-β21S sample, rendered transparent to reveal the grain boundary network within. The grains from the LabDCT analysis are plotted as cubes at their measured positions, revealing their (relative) size and crystallographic orientation (by colour). (**b**) (100) pole figure calculated from the grain orientations. The colour scale represents multiples of a random distribution.

Figure 2(a) shows a grain map reconstructed from a LabDCT scan of the Ti-β21S sample. Each grain is represented by a cube showing its centroid position and the unit cell orientation of its lattice in relation to the absorption reconstruction of the sample. The crystallographic orientation of the grains and also their size are illustrated in relative terms in Fig. 2(a) by the width of the cube representing the grain radius. The pole figure calculated from these grain orientations (Fig. 2(b)) indicates weak crystallographic texture, close to random.

### Measurements

A specific set of measurements were performed in order to validate the information gained from the LabDCT reconstruction, both the spatial location of grains and the grain orientations. EBSD analysis was performed on a cross-section of grains in the sample in order to compare against the grain orientations measured using the LabDCT technique. Synchrotron and laboratory propagation phase contrast tomography (PCT) measurements were performed and used to independently confirm the validity of the grain positions (centre’s of mass) and sizes. The PCT reconstructions are sensitive to the alpha phase located at the grain boundaries. This represents the ‘perfect’ case in that the 3D grain shapes, and thus individual grain volumes in the sample, can be imaged directly. In the following we evaluate the grain orientation and spatial location measurements in turn.

### Validation of grain orientation measurement

**Figure 3:**
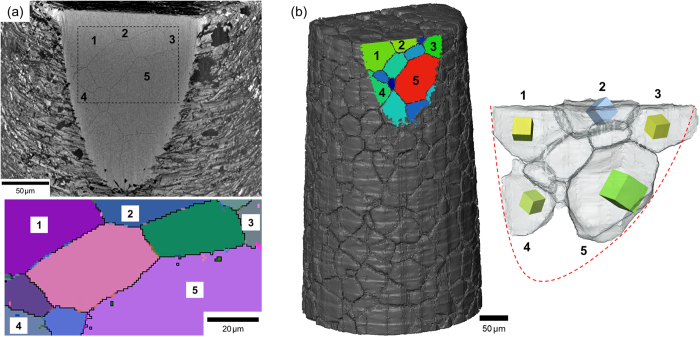
(**a**) Top: SEM image showing the grains in a cut section of the sample used for EBSD analysis. Below: EBSD map of the region indicated in the SEM image showing the grain orientations. The grain numbers 1 to 5 are used for validation against the corresponding grains measured in LabDCT. (**b**) Left: synchrotron PCT reconstruction of the sample showing the corresponding grains in the cut section. Right: The individual grains 1 to 5, rendered transparent, revealing the corresponding cubes from the LabDCT within. The dashed outline represents the boundary of the cut section.

The grain orientations measured from the LabDCT reconstruction were compared against the well-established electron backscatter diffraction (EBSD) method for determining grain orientation. A small section was cut from the top of the sample to reveal several grains, illustrated in the scanning electron microscope (SEM) micrograph of Fig. 3(a). EBSD analysis revealed the orientations of the grains on this cut surface. By aligning the LabDCT absorption mask and the synchrotron PCT dataset each grain in the LabDCT reconstruction was matched to a corresponding grain in the PCT reconstruction (within the catchment defined by the grain boundary structure). In this way the grains measured by LabDCT lying on the cut surface were matched with the PCT and labeled 1–5 in Fig. 3. Note, the four smaller grains in the middle of this group of five were not detected by LabDCT and are thus not available for validation with EBSD. In order to compare the orientation accuracy of grains between EBSD and LabDCT the misorientation between pairs of grains within this group of five grains was calculated and is shown in Table 1. The EBSD measurements are expected to have an orientation accuracy in the order of a few tenths of degrees, and the observed differences are within this range. The LabDCT method can potentially provide higher orientation accuracy, since the geometric conditions and accuracy are very similar to those used and demonstrated by strain measurements with synchrotron-based DCT.

### Evaluation of LabDCT grain tracking

**Figure 4:**
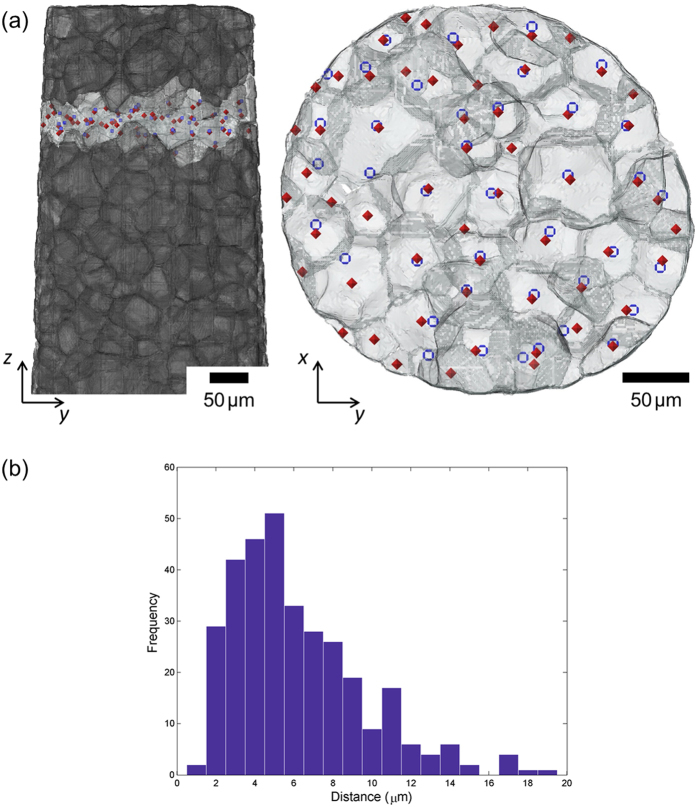
(**a**) A ‘layer’ of grains (right) showing the grain boundaries determined by PCT with their respective centers of mass from the PCT reconstruction (red dots) compared with the centers of the corresponding matched grains measured with LabDCT (blue circles). The position of this grain layer relative to the sample height is indicated (left). (**b**) Frequency distribution of the distances between PCT and LabDCT centroids for all the matched grains in the whole sample, with a mean distance of 7.1 μm.

**Figure 5:**
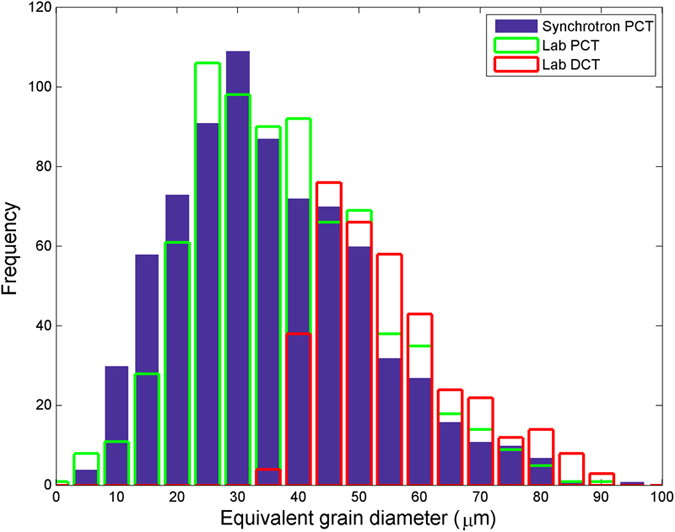
Equivalent grain diameters, measured from the same sample volume from the synchrotron PCT, laboratory PCT and laboratory DCT reconstructions.

In order to assess the ability of LabDCT to measure grain sizes and grain locations, the LabDCT measurements are compared against synchrotron PCT. A small fraction of the grain boundaries (often small angle grain boundaries) in the PCT volume could not be segmented correctly due to no alpha phase being precipitated, which may have resulted in a few erroneous grain centroids. In Fig. 4(a), the positions of the grain centroids from the PCT dataset are compared to the positions of the centre’s of mass of the corresponding matched grains measured from the LabDCT scan for a single ‘layer’ of grains within the sample. It can be seen that the majority of grains in this layer, indeed 40 out of 58, have a matching grain in the LabDCT reconstruction. Most of the undetected grains seem to lie at the surface where the sample has been machined from a larger piece of the material leaving smaller grains. The histogram in Fig. 4(b) shows that the mean difference in the location of the grains between PCT and LabDCT is 7.1 μm (with a standard deviation of 4.3 μm) or 4.2 voxels (standard deviation of 2.5 voxels). The histograms in Fig. 5 clearly show that LabDCT accurately captures the larger grains but misses the grains below 40 μm. Consequently, the average grain size for LabDCT (58 μm from 378 measured grains) is larger than for laboratory or synchrotron PCT (38 μm from a total of 853 grains. This current minimum detectable size for LabDCT is consistent with the four smaller grains being missed in Fig. 3(b). Roughly half the grains by number or 83% by volume are detected by LabDCT in this case. The scattered intensity or signal strength is proportional to the grain volume and to the third power of the grain radius. The grain size detection limit is determined by the signal to background noise in the diffraction area of the detector, and it is around 40 μm in the current scan.

### Comparison of PCT and LabDCT grain reconstructions

**Figure 6:**
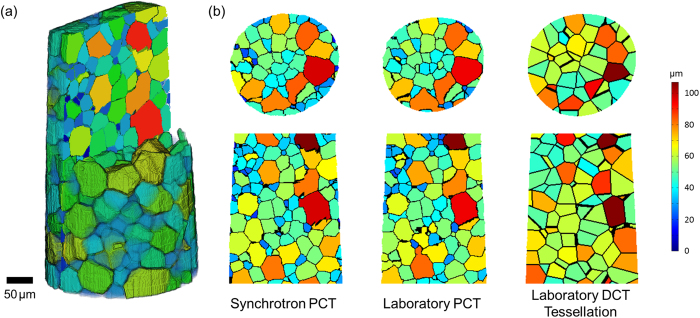
(**a**) Non-destructive representation of grain morphology by a virtual slice through the sample volume. (**b**) Comparing grain shapes of the sample extracted from reconstructed volumes acquired using three modalities: synchrotron phase contrast, laboratory phase contrast and laboratory diffraction contrast tomography. In each case the grains are shown mapped to a colour scale based on grain size.

**Figure 7:**
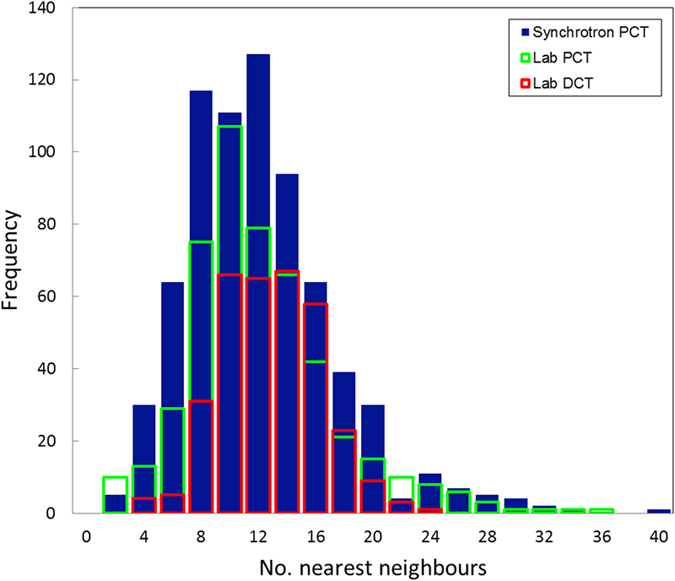
Number of nearest, touching neighbors for each grain in the sample, calculated from the synchrotron PCT, laboratory PCT and tessellated laboratory DCT reconstructions.

The same region of sample has been imaged using three modalities: synchrotron PCT, laboratory PCT and laboratory DCT. The two PCT measurements have used the contrast arising from the grain boundary alpha phase in order to reconstruct the 3D grain shapes. For the LabDCT measurement the centres of mass and size of the grains are obtained. In the current version of the LabDCT approach using Laue focusing no attempt is made to reconstruct their 3D shape utilizing the shape information embedded in the diffraction signals. Instead, a Laguerre tessellation method has been applied, using the LabDCT data, to create a volume of polyhedra representing the individual crystal grains^24^. The grain centres are used as a distribution of distinct generating points or seeds to divide the absorption mask of the sample into corresponding regions in 3D. The relative sizes of these 3D regions are inferred from the grain volumes measured with LabDCT. In Fig. 6 sections through the tessellated dataset of the sample are shown, together with equivalent sections through the two PCT reconstructions. The grains are coloured according to their size in each case. In the case of the tessellated dataset the grain size measured from the LabDCT grain reconstruction is used (rather than the size of the subsequently computed polyhedra). It can be seen that the laboratory PCT reconstruction matches well with the synchrotron PCT with good correspondence in the positions of large and small grains. There exist a few regions in the laboratory PCT volume where a grain boundary is not reconstructed resulting in the segmentation of what is actually two neighbouring grains as a single grain. This is likely to explain the observation in Fig. 5 in the grain size distribution of the laboratory PCT reconstruction that fewer grains below 20 μm are measured compared to the synchrotron PCT. Regards the LabDCT tessellation it can be seen that the location of the larger grains, those above 70 μm for example, correspond well with the PCT reconstructions. The grain size measured from LabDCT is slightly over-estimated; indeed the distribution in Fig. 5 lies slightly above that of both PCT reconstructions at all sizes above 50 μm. The computation of a tessellated space filling volume of the grains has enabled a comparison of the distributions of nearest neighbours, shown in Fig. 7. The two PCT reconstructions have very similar distributions (average of 11.6 nearest neighbours) while that for the LabDCT is slightly higher (~12.4). As evident from Fig. 7, the two PCT analyses have grains with more than 24 neighbours (the LabDCT analysis does not have any), while Fig. 5 shows a significantly greater number of smaller grains in the PCT volumes. Small grains can give rise to both fewer small grain neighbours when surrounded by a big grain, and to many neighbours as the number of counts (of small grains) increases for larger grains.

## Discussion

The new laboratory X-ray diffraction contrast tomography module GrainMapper3D™ and the measurement technique has been described and its capability demonstrated by reconstructing the crystal grains within a polycrystalline titanium alloy sample. The crystallographic orientation and spatial location of the grains are validated with electron backscatter diffraction measurements and phase contrast tomography reconstruction of the grain structure respectively. The approach uses a specific Laue focusing geometry allowing multiple hundreds of grains, and potentially approaching one thousand, to be mapped in a single scan. Currently, the minimum detectable grain size is around 40 μm. This value is primarily influenced by the scattering power of the material, the flux density of the X-ray source and the sensitivity of the X-ray detector system. Further work on improving the minimum detectable grain size is ongoing.

The capability to link directly the crystallographic and grain microstructure information with that obtained via conventional absorption or phase contrast imaging, non-destructively in three-dimensions and all in the laboratory, creates a powerful tool with the potential to revolutionize the way in which 3D materials science investigations are conducted. A significant attraction is the ability to track changes, due to mechanical load or temperature for example, over time during repeated observation (e.g. ‘4D’ studies). The inherent non-destructive nature of the technique provides a unique opportunity for direct coupling of 3D/4D experimental data with materials simulations, significantly enhancing our understanding of current materials performance and enabling the design and exploration of new materials. Where the extent of deformation precludes the reconstruction of a grain map via the LabDCT method, the relationship between the initial crystallographic and grain microstructure and subsequent damage mechanisms within the bulk of polycrystalline materials can be investigated. Application to 3D crystal plasticity modeling will enable numerical simulations of damage and deformation behaviour on as-measured 3D polycrystalline microstructures to be performed^25^. Crystal plasticity FE (CPFE) simulations have been combined with Voronoi tessellation methods to generate artificial microstructures and applied to studies of nanoindentation^26^, fatigue^27^,^28^ and twinning^29^,^30^. The spatial position and orientation of grains acquired from a reconstructed grain map of a bulk polycrystalline sample can be used as direct input to a finite element mesh. Furthermore, comparison between simulation and experimentally observed deformation behaviour can aid improvement of such models, and ultimately guide materials performance. Recrystallization and grain growth processes that can occur during annealing of a metal or alloy have important implications for the properties of the material.

The technique described here can enable the realization of all the mentioned examples for routine studies in the laboratory by allowing direct observation of microstructure evolution during grain growth and the measurement of dynamics of individual grains. Direct comparison to theory/models can be performed (e.g^31^.) without having to rely on statistical measures of grain size distribution, etc. Furthermore, correlation between grain orientation and growth of grains and the relationships between neighbouring grains can be explored. While no attempt is made in the current version of the LabDCT approach, the potential exists to reconstruct 3D grain shapes by collecting projection information of the diffracting crystal grains. Imaging in the projection plane, at a distance further from the sample than the Laue focal plane, can give access to shape information embedded in the diffraction signals. Knowledge of exact grain morphology would enable detailed information of nearest neighbor grain interactions to be obtained, further enhancing in-depth studies of grain growth.

## Methods

### Material

The material used to demonstrate the technique is a metastable beta titanium alloy Tiβ21S (Ti–15Mo–3Nb–3Al–0.2Si–0.2Fe) from which a sample of 300 μm diameter was machined. The alloy microstructure consists of equiaxed grains of the metastable beta phase, having a body-centred cubic (bcc) lattice. The sample was heat treated at a temperature of 830 °C for 30 min in order for grain growth to occur, giving an average grain size of ~40 μm. A further heat treatment of 15 min at 725 °C was applied to cause a thin layer of the hexagonal (hcp) alpha phase to be precipitated on the grain boundaries. The alpha phase is enriched with Al, while the beta phase is enriched with Mo and Nb. This results in a detectable difference in electron density between α and β phases, sufficient that the grain boundaries can be visualised in 3-D using phase contrast tomography (PCT).

### EBSD measurements

The EBSD analyses were conducted on a Zeiss Sigma HDVP at 20 kV accelerating voltage, 8 nA beam current and 5 mm working distance with a 0.82 um step size. The data were collected using the Oxford Instruments NordlysMax^2^ EBSD detector and AZtecHKL software. The sample was initially prepared via FIB polishing using 240 pA current at 5 kV.

### PCT measurements

A tomographic reconstruction of the sample was produced from propagation-based synchrotron X-ray PCT data acquired on beamline ID19 at the European Synchrotron Radiation Facility (ESRF). 800 projection images were acquired with a pixel size of 0.56 μm, using an energy of 35 keV and a sample–detector distance of 25 mm. Propagation-based laboratory X-ray PCT was performed using a ZEISS Xradia 520 Versa system from Carl Zeiss X-ray Microscopy. The sample was placed at a distance of 11 mm from the source, with the detector 120 mm from the sample. Using a detector objective lens giving an optical magnification of 4 and a binning mode of 2 × 2, this resulted in an effective pixel size of 0.57 μm. An accelerating voltage of 70 kV and a current of 86 μA gave a transmission through the sample of ~26%. 2900 projection images were acquired around a sample rotation of 360°, with an exposure time per image of 30 s.

### LabDCT measurements

The LabDCT measurements were conducted on a ZEISS Xradia 520 Versa instrument equipped with a GrainMapper3D™ analysis package. The source–sample and sample–detector distances were both set to 12 mm, using the 1:1 distance ratio of the source and detector to benefit from the Laue focusing effect. This geometric magnification provided an effective pixel size of 1.7 μm. The same X-ray source settings were used as for the PCT scan: accelerating voltage 70 kV and current 86 μA. For the LabDCT scan 180 diffraction pattern images were acquired around a 360° rotation of the sample in steps of 2° with an exposure time per image of 300 s. 1000 projection images each of 2.5 s exposure were acquired for the absorption contrast scan for reconstruction of the absorption mask.

In order to find and confirm the same grains from the LabDCT scan as those revealed on the cut surface of the sample used for EBSD analysis, the synchrotron phase contrast tomography (PCT) reconstruction of the sample was scaled (under-sampled by a factor of 3) to give the same effective voxel size as the absorption mask reconstruction of the LabDCT scan, and the two datasets were aligned. The plane defined by the cut surface was observable in the absorption mask volume and thus was used to virtually cut the PCT volume of the sample in the same position, thus revealing the grains. The grain shapes in the SEM image and in the virtual section of the PCT volume match well as highlighted in Fig. 3(b).

## Additional Information

**How to cite this article**: McDonald, S. A. *et al*. Non-destructive mapping of grain orientations in 3D by laboratory X-ray microscopy. *Sci. Rep*. **5**, 14665; doi: 10.1038/srep14665 (2015).

## Figures and Tables

**Table 1:** Comparison of misorientation angles (in degrees) between grain pairs as measured by EBSD and LabDCT.

Grain No.	EBSD	LabDCT	Difference
1→2	53.5	53.2	0.3
1→3	52.5	52.7	0.2
1→4	56.9	57.4	0.5
1→5	49.7	50.1	0.4
2→3	52.5	52.4	0.1
2→4	51.6	51.3	0.3
2→5	29.0	28.9	0.1
3→4	9.1	9.5	0.4
3→5	41.3	41.4	0.1
4→5	43.8	44.0	0.2

The mean difference in misorientation is 0.26°.
